# Integrated Chemical Interpretation and Network Pharmacology Analysis to Reveal the Anti-Liver Fibrosis Effect of *Penthorum chinense*

**DOI:** 10.3389/fphar.2022.788388

**Published:** 2022-06-02

**Authors:** Zenan Du, Doudou Huang, Pengjie Shi, Zhiying Dong, Xiujuan Wang, Mengshuang Li, Wansheng Chen, Feng Zhang, Lianna Sun

**Affiliations:** ^1^ School of Pharmacy, Shanghai University of Traditional Chinese Medicine (SHUTCM), Shanghai, China; ^2^ Institute of Chinese Materia Madica, Shanghai University of Traditional Chinese Medicine, Shanghai, China; ^3^ Department of Pharmacy, Changzheng Hospital, Second Military Medical University, Shanghai, China

**Keywords:** DDA-assisted DIA, network pharmacology, action of mechanism, *Penthorum chinense*, liver fibrosis

## Abstract

Liver fibrosis is a disease with complex pathological mechanisms. *Penthorum chinense* Pursh (*P. chinens*e) is a traditional Chinese medicine (TCM) for liver injury treatment. However, the pharmacological mechanisms of *P. chinens*e on liver fibrosis have not been investigated and clarified clearly. This study was designed to investigate the chemicals in *P. chinens*e and explore its effect on liver fibrosis. First, we developed a highly efficient method, called DDA-assisted DIA, which can both broaden mass spectrometry (MS) coverage and MS^2^ quality. In DDA-assisted DIA, data-dependent acquisition (DDA) and data-independent acquisition (DIA) were merged to construct a molecular network, in which 1,094 mass features were retained in *Penthorum chinense* Pursh (*P. chinens*e). Out of these, 169 compounds were identified based on both MS^1^ and MS^2^ analysis. After that, based on a network pharmacology study, 94 bioactive compounds and 440 targets of *P. chinens*e associated with liver fibrosis were obtained, forming a tight compound–target network. Meanwhile, the network pharmacology experimental results showed that multiple pathways interacted with the HIF-1 pathway, which was first identified involved in *P. chinens*e. It could be observed that some proteins, such as TNF-α, Timp1, and HO-1, were involved in the HIF-1 pathway. Furthermore, the pharmacological effects of *P. chinense* on these proteins were verified by CCl_4_-induced rat liver fibrosis, and *P. chinense* was found to improve liver functions through regulating TNF-α, Timp1, and HO-1 expressions. In summary, DDA-assisted DIA could provide more detailed compound information, which will help us to annotate the ingredients of TCM, and combination with computerized network pharmacology provided a theoretical basis for revealing the mechanism of *P. chinense*.

## Introduction

Traditional Chinese medicine (TCM) has been used by the Chinese community for treating various diseases for more than 2,000 years, and it is an important source for modern drug development (Liu et al., 2017). In recent years, the method of integrated pharmacology and biological networks has been applied to fields in the life sciences (Ding et al., 2021). Among them, based on drug-target network construction and analysis of network characteristics, the methods of multiple target overall control are gradually being used to predict the main active ingredients and potential target groups of TCM and to define the mechanisms by which TCMs exert curative effects (Zhou et al., 2018). However, due to its complex components, it is hard to clarify the mechanism of TCM, which is a main obstacle for acceptance internationally. Thus, understanding the chemical components in TCM will be a benefit for clarifying its mechanism since chemicals are the key substances that exert drug effects. Traditional chemical interpretation includes isolation and identification, which is time-consuming. In addition, splitting TCM into individual compounds violates the essence of TCM as a whole. Therefore, comprehensively characterizing the chemical components of TCM is helpful to clarify the material basis for its efficacy. Liquid chromatography–mass spectrometry (LC-MS) analysis can quickly and accurately describe the chemical components in TCM. In previous studies, various data post-processing technologies have been invented to comprehensively identify herbal components in complex sample matrices, such as MDF or EIC (Pan et al., 2018; Wang et al., 2021a; Dai et al., 2021). Data-independent acquisition (DIA) provides comprehensive untargeted acquisition of molecular data, while data-dependent acquisition (DDA) displays a signal detected in a given sample. Due to the insufficient detection coverage and low sensitivity of DDA and DIA modes, we proposed a technology that combined DDA and DIA for characterizing the chemical composition of TCM effectively. After that, network pharmacology was used to predict the potential mechanism of TCM. In this study, we used *P. chinense* as a case to illustrate the procedure of an integrated study of chemical interpretation and network pharmacology in TCM.

*Penthorum chinense* is a traditional herbal medicine of the Miao nationality, often used to treat various liver diseases, and can be used as vegetables or energy drinks (Wang et al., 2020). Clinically, gansu granules, the single herb prescription preparation of *P. chinense*, are often used to treat jaundice, cholecystitis, nonalcoholic and alcoholic fatty livers, and infectious hepatitis (He et al., 2019; Wang et al., 2020). Based on the great clinical effects of *P. chinense*, the chemical components that exert the hepatoprotective effect and the mechanism of its curative effect have been widely explored. Previously, our research group made a systematic study on the chemical components of *P. chinens*e in the early stage and found that flavonoids, phenolic acids, and lignans were the main constituents of *P. chinense*. Through *in vitro* experiments, it was proved that the polyphenols and flavonoids in *P. chinense* have a good effect on anti-liver fibrosis (Huang et al., 2014; Huang et al., 2015a).

**FIGURE 1:**
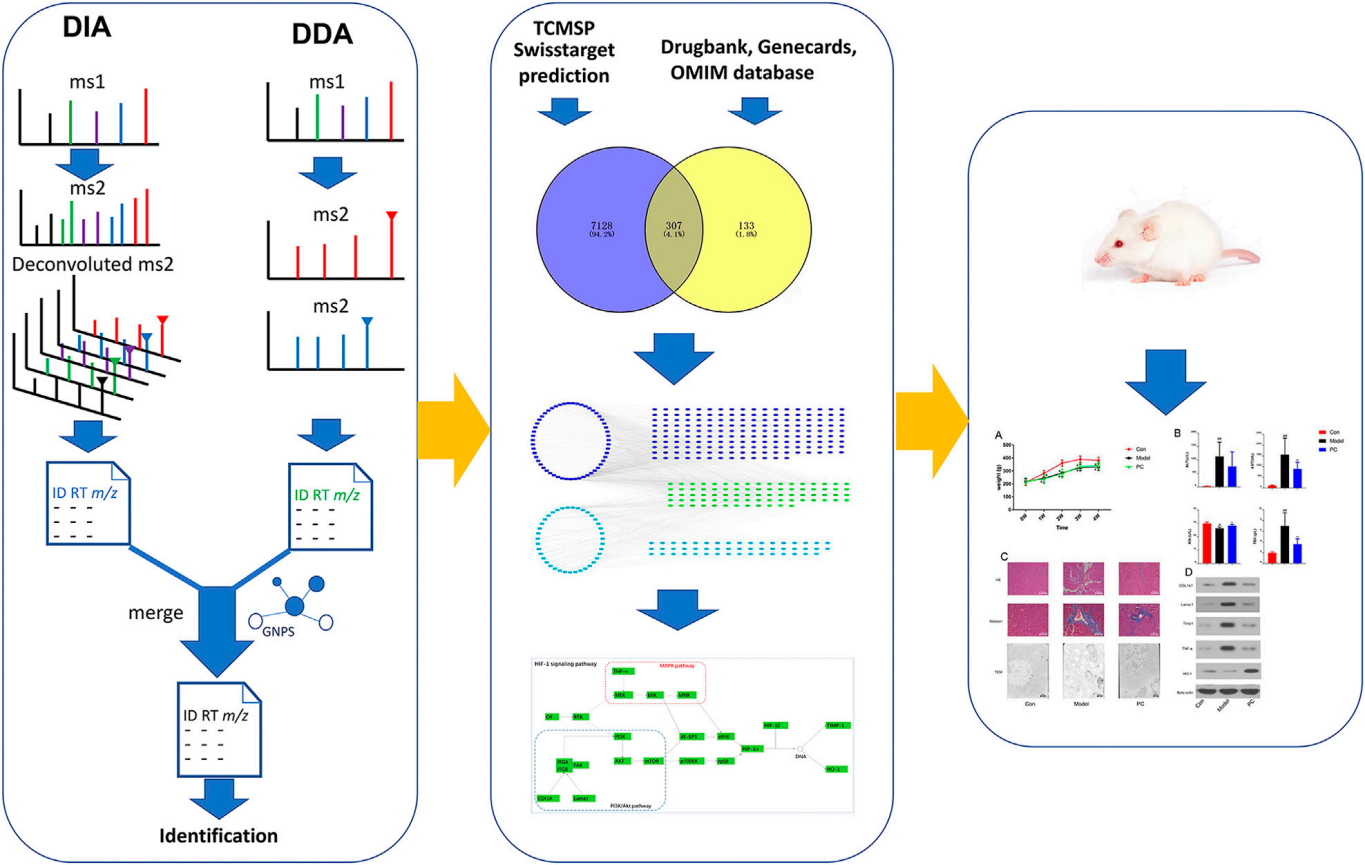
Strategy workflow for this study design.

As a continuous study, we proposed an LC-MS method, combining DDA and DIA, for comprehensively characterizing the components in *P. chinense*. Then, network pharmacology was used to predict the mechanism of *P. chinense*, which was confirmed by *in vivo* experiments (Figure 1). All aforementioned schemes might provide a new strategy for TCM chemical and mechanism study.

## Materials and Methods

### Chemicals and Reagents

Carbon tetrachloride (CCl_4_) and formic acid were from Sigma-Aldrich (Darmstadt, Germany). Methanol and acetonitrile (ACN) were from Merck (Darmstadt, Germany). Sprague–Dawley (SD) rats were from the Shanghai Slac laboratory animal company (Shanghai, China). The reference 38 standards of vanillic acid, gallic acid, ethyl gallate, pinocembrin, apigenin, kaempferol, luteolin, ferruginol, brevifolin carboxylic acid, ellagic acid, quercetin, ethyl brevifolincarboxylate, 2′,4′,6′-trihydroxyacetophenone 4′-O-β-glucoside, kaempferol-3-O-arabinoside, pinocembrin-7-O-β-D-glucoside, 2′,4′,6′-trihydroydihychalcone-4′-β-D-glucoside, 4-deoxy-phlorizin, kaempferol-3-O-α-L-rhamnoside, alpinetin-7-O-β-D-glucoside, quercetin-3-O-α-L-arabinoside, (-)-epicatechin-3-O-gallate, quercitrin, isoqueritrin, (E)-phenylpropene-3-methoxyphenyl-(6″-O-galloy)-4-O-β-D-glucoside, penthorumin A, penthorumin B, 2′,6′-dihydroydihychalcone-4′-O-(3″-O-galloy)-β-D-glucoside, penthorumin D, rutin, quercetin-3-O-β-D-glucosyl-(1→2)-β-D-glucoside, 2′,4′,6′-trihydroxyacetophenone-4′-O-[4.6-(R)-HHDP]-β-glucoside, 2′,4′,6′-trihydroxyacetophenone-4′-O-[4.6-(S)-HHDP]-β-glucoside, β-1,4,6-tri-O-galloyl-D-glucoside, pinocembrin-7-O-[4″,6’’-(S)-hexahydroxydiphenoyl]-β-D-glucoside, pinocembrin-7-O-[3′-O-galloyl-4′,6’-(S)-hexahydroxydiphenoyl]-β-D-glucoside, and 2′,6′-dihydroxydihydro-chalcone-4′-O-(3″-O-galloyl-4″,6″-hexahydroxydiphenoyl)-β-D-glucoside, Penthorin B and (7′E)-2′,4,8-trihydroxy-3-methoxy-2,4′-epoxy-8,5′-neolign-7′-en-7-one were all isolated from *P. chinens*e in our previous experiment. The purities of all references were over 98%.

### Chemical Characterization Analysis of *Penthorum chinense* Using Liquid Chromatography to Quadrupole/Time-of-Flight Mass Spectrometry

#### Sample Preparation

*Penthorum chinense* was purchased from Gulin, Sichuan Province, China, in July 2017. The plant was identified and deposited at the Shanghai University of Traditional Chinese Medicine (PC20170618). Briefly, *P. chinense* (2 kg) was refluxed with 80% ethanol (20 L) twice (2 h each time); the combined solution was condensed under vacuum conditions at 60°C and yielded a crude extract (245 g, extract ratio = 12.5%). The concentrated extracts were stored at −4°C and sealed for later use *in vivo*.

#### LC-Q-TOF-MS Analysis

The extracts (10 mg) were dissolved in 80% methanol (25 ml) and centrifuged at 10,000 rpm for 20 min, and the supernatants were subject to LC-MS analysis. Ultra-high-pressure liquid chromatography (UHPLC) separation was performed on an Agilent UHPLC system (Agilent Technologies, Santa Clara, CA, United States) and a Waters ACQUITY UPLC BEH C18 column (2.1 mm × 100 mm, 1.7 µM). The UHPLC system used 0.1% formic acid in water as phase A and acetonitrile as phase B. The optimized elution program was as follows: 0–15 min (5%–30% B), 15–25 min (30%–40% B), 25–33 min (40%–95% B), and 33–35 min (95% B). The column temperature was maintained at 40°C, while the auto-sampler was maintained at 4°C. 4 μl of each sample was injected and a flow rate of 0.3 ml/min was applied during the gradient elution.

MS analysis was performed in the negative mode under the following parameters: scan range, *m/z* 100–1,700, acquisition rate/time: 3.00 spectra/s, gas temp: 350°C, drying gas: 11 L/min, nebulizer: 45 psig, sheath gas temp: 350°C, sheath gas flow: 11 L/min, Vcap: 3500 V, nozzle voltage: 500 V, fragmentor: 140 V, Skimmer: 65 V, and CE: 0 and 30 V.

#### Raw Data-Preprocessing Parameters

The MS-Dial software (version 4.24 https://prime.psc.riken.jp/compms/msdial/main.html) was used to automatically extract targeted ions. The parameters were as follows: MS1 tolerance, 0.01 Da; MS^2^ tolerance, 0.025 Da; retention time range, 0.5–35 min; MS^1^ mass range, 100–1,000; MS/MS mass range, 0–1,000; minimum peak height, 2,000 amplitude; mass slice width, 0.1 Da; sigma window value, 0.5; MS/MS abundance cutoff, 500 amplitude; retention time tolerance, 0.05 min.

#### Molecular Networking

The Global Natural Products Social Molecular Network (GNPS) is an online platform in which targeted compounds could easily link with those untargeted compounds having similar MS/MS spectra (Pan et al., 2020); then, the potential unknown compounds could be quickly identified based on their similar MS/MS spectra to the target compound (Yu et al., 2021). The corresponding molecular networking was created according to the online workflow at GNPS (http://gnps.ucsd.edu) with a parent mass tolerance of 0.02 Da, an MS/MS fragment ion tolerance of 0.02 Da, and the minimum cluster size of 1; run MScluster and filter precursor window tools were turned off. The network was created with a cosine score above 0.7 and more than four matched peaks. The molecular networking data were visualized using Cytoscape (ver.3.7.2).

### Candidate Compound Screening

All the identified chemicals in *P. chinense* were submitted to the Traditional Chinese Medicine Systems Pharmacology Database and Analysis Platform (TCMSP, http://tcmspw.com) for compound screening, which should meet the criteria of oral bioavailability (OB) ≥30% and drug-likeness (DL) ≥0.14 as candidate compounds for further analyses. OB is an important parameter to measure the pharmacokinetics and druggability of drugs *in vivo*. It represents the convergence of the processes of absorption, distribution, metabolism, and excretion (ADME) (Williams et al., 2022). DL is a qualitative principle used in drug design to accurately predict the “drug-like” nature of a compound (Bitew et al., 2021).

#### Identification of Drug Targets

To obtain as many compound targets as possible, we searched and predicted targets from TCMSP (https://www.tcmsp-e.com/) and Swiss Target Prediction (http://www.swisstargetprediction.ch/). All targets were restricted to human origin. Next, we retrieved the protein targets of hepatic fibrosis/liver fibrosis from several databases, such as the DrugBank (https://www.drugbank.ca/), GeneCards (https://www.genecards.or), and OMIM (https://omim.org/). Then, all target names were converted into the corresponding official gene names in the UniProt database. The Venny2.1.0 online tool (http://bioinfogp.cnb.csic.es/tools/venny/index.html) was used to obtain the overlapping targets from the two sources to identify potential drug targets for the treatment of liver fibrosis.

#### Gene Ontology Enrichment Analysis of Targets

To systematically understand the biological processes of *P. chinense* in the treatment of liver fibrosis, we performed gene ontology (GO) enrichment analysis of potential targets. The terms with a *p*-value of less than 0.05 were selected for functional annotation and signaling pathway clustering. The above analysis was completed using the functional annotation tool of Metascape (https://metascape.org/gp/index.html#/main/step1).

#### Network Construction and Analysis

To comprehensively analyze the molecular mechanism of *P. chinense* in the treatment of liver fibrosis, a compound–target–pathway network was constructed using Cytoscape 3.7.1 software. The NetworkAnalyzer tool in the software was used to analyze the network topology properties.

### Experiment Design and Drug Administration

Male SD rats weighing 250 ± 20 g were purchased from the Center of Experimental Animals at the Shanghai Slac Laboratory Animal Co., Ltd. (SCXK (hu) 2018-0016, Shanghai, China). The animal experiments were approved by the Ethics Committee of the Second Military Medical University (Shanghai, China). All animal procedures were in accordance with the National Institutes of Health Guide for the Care and Use of Laboratory Animals. All rats were housed at a standard room temperature of 23 ± 2°C and a humidity of 55%–70% under a 12 h light/dark cycle with access to food and water *ad libitum*.

After a week of adaptive feeding, SD rats were randomly divided into two groups: the control group and the model group. Model group rats were intraperitoneally injected with 40% CCl_4_ (3 ml/kg in olive oil) for 4 weeks, once every 3 days. Simultaneously, the model rats were randomly divided into two groups (six rats in each group): 1) model group: model rats treated with distilled water and 2) PC: the *P. chinense* extract was dissolved in distilled water (0.33 g/ml), and SD rats were administrated with the *P. chinense* extract 10 ml/kg per day for 4 weeks (3.3 g/kg/d). Normal rats were treated with the same volume of distilled water.

#### Histopathological Examination

All rats were sacrificed after 4 weeks of treatment, and the left lobe (2 cm × 2 cm) of the liver of three groups was immobilized in paraformaldehyde. After that, the liver tissue was dehydrated, embedded in paraffin, and then cut into sections for hematoxylin and eosin (HE) and Masson staining. The formation and change of egg granuloma were observed under a light microscope. The Masson stain section was examined for changes in collagen deposition, which showed blue staining areas.

#### Transmission Electron Microscopy

The right lobe of the liver [1.5 mm^3^] of three groups was immobilized in 4% glutaraldehyde for 2 h, followed by immobilization in 1% osmium tetroxide for 2 h. After that, the liver tissue was dehydrated with acetone and embedded in 618 epoxy resins. All the fixed biopsies were observed under a Hitachi H-600 transmission electron microscope.

#### Biochemical Analysis

The serum concentrations of aspartate aminotransaminase (AST, C010-2-1), alanine aminotransferase (ALT, C009-2-1), albumin (Alb, A028-2-1), and total bilirubin (TBil, C019-1-1) were evaluated using a detection kit (Nanjing Jiancheng Bioengineering Institute, Nanjing, China) according to the manufacturer’s instructions. For AST and ALT analysis, 5 µl of serum or the blank was incubated with 20 µl of the matrix reagent for 30 min at 37°C; then, 20 µl of the chromogenic reagent was added for 30 min at 37°C, followed by adding 200 µl of the termination reagent. The absorbance at 510 nm was measured using a spectrophotometer (Synergy HT, BioTek Instrument Inc., Winooski, VT, United States). For Alb analysis, 2.5 µl of serum or the blank was incubated with 250 µl of the reagents for 10 min at room temperature, and the absorbance at 628 nm/630 nm was measured using a spectrophotometer (Synergy HT, BioTek Instrument Inc., Winooski, VT, United States). For TBil analysis, 8 µl of serum or the blank was incubated with 240 µl of reagent I for 5 min at 37°C, followed by adding 60 µl of reagent II for 5 min at 37°C, and the absorbance at 450 nm was measured using a spectrophotometer (Synergy HT, BioTek Instrument Inc., Winooski, VT, United States).

#### Western Blot Analysis

Western blot analysis was performed according to standard procedures. In brief, tissues were lysed in Tissue Protein Extraction Reagent (T-PER) and were then centrifuged at 10,000 rpm for 5 min to remove the insoluble matter. The protein concentration was determined using a BCA protein concentration assay kit (Pierce BCA Protein Assay Kit). Equal amounts of the protein (100 μg) were separated via 10% sodium dodecyl sulfate–polyacrylamide gel electrophoresis and transferred to a PVDF membrane (IPVH00010, Germany). The membrane was incubated with 5% skim milk in Tris-buffered saline–Tween 20 to block nonspecific binding sites and incubated with the primary antibody overnight at 4°C. The antibodies used were as follows: anti-COL1A1 (catalog # sc-28657, 1:800, Santacruze), anti-Lama-1 (catalog # sc-5582, 1:500, Santacruze), anti-Timp1 (catalog #9496, 1:300, Santacruze), anti-TNF-α (catalog # sc-1351, 1:500, Santacruze), anti-HO-1 (catalog # sc-10789, 1:400, Santacruze), and anti-β-actin (catalog # SC-47778, 1:800, Santacruze) were purchased from Santacruze (Dallas, TX, United States). Peroxidase-conjugated anti-rabbit (1:3,000), anti-mouse secondary antibodies (1:5,000), and anti-goat secondary antibodies (1:2,000) were used as secondary antibodies. ECL reagent (Chemiluminescent Substrate, Thermo, United States) was used to detect antigen–antibody complexes. The protein expression levels were normalized to that of β-actin in the same sample.

## Results

### Schematic Workflow of DDA-Assisted Data-Independent Acquisition

**FIGURE 2:**
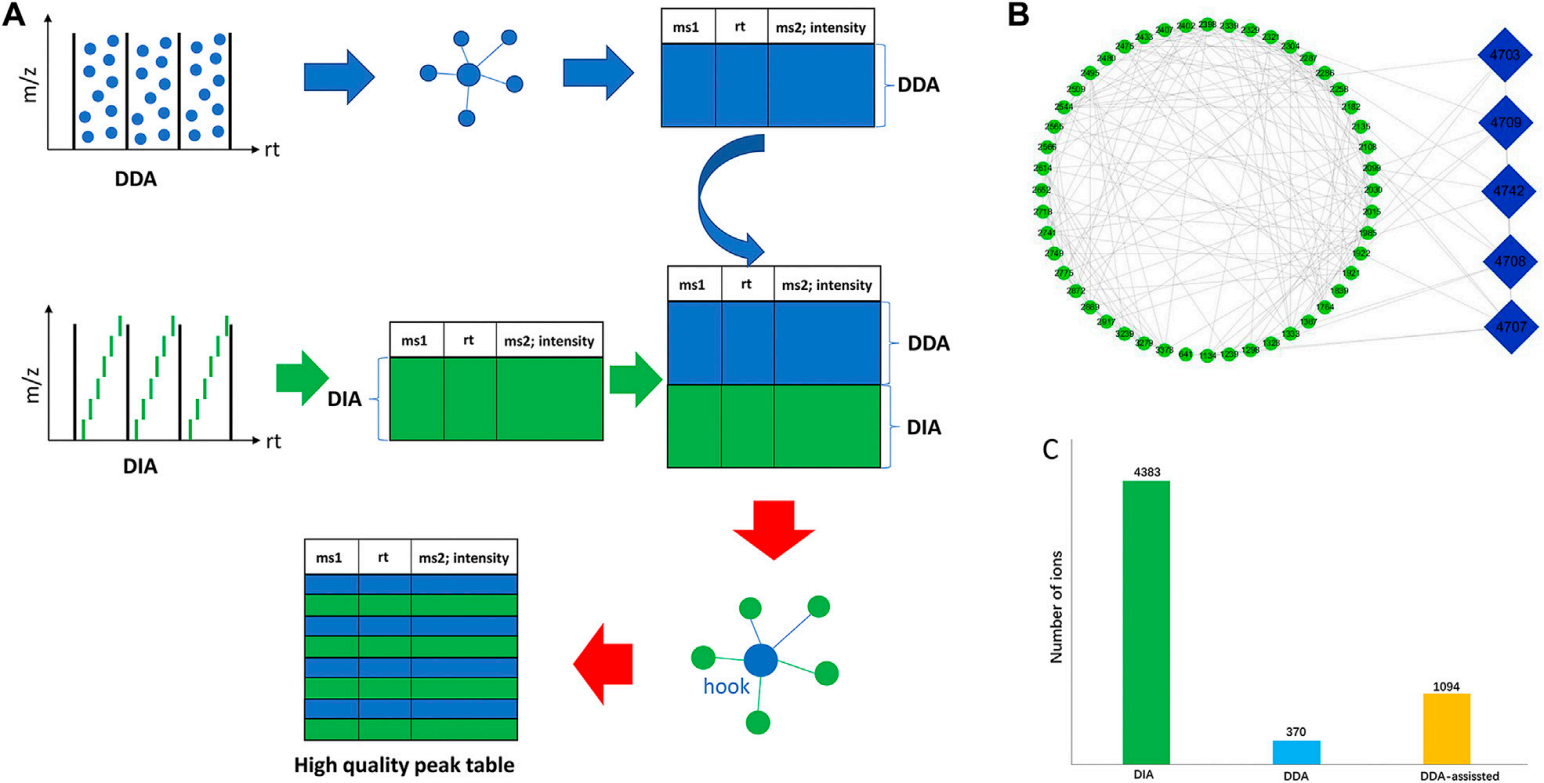
**(A)** Overall workflow of DDA-assisted DIA analysis. **(B)** Specific illustration of DDA-assisted DIA. The green ellipse represents the DIA MS features, and the blue diamond represents the DDA MS features. The edges were produced by the combination of similar MS^2^ features of DDA and DIA through GNPS analysis. **(C)** Summary of MS features through DIA, DDA, and DDA-assisted DIA.

In the LC-MS analysis of traditional Chinese medicine, thousands of ion signals are often generated, so the identification of components in traditional Chinese medicine has always been a difficulty in the research. Although it is considered that the comparison with the reference substance is the most accurate method for identification, the components of traditional Chinese medicine are very complex and it is difficult to obtain a more perfect reference substance. Therefore, the chemical component information in traditional Chinese medicine can be accurately clarified by using ion fragment analysis combined with the decomposition law of chemical components. At present, there are two common acquisition modes in high-resolution mass spectrometry that can obtain MS^1^ and MS^2^ information at the same time: the data-dependent acquisition (DDA) mode and data-independent acquisition (DIA). Commonly used DIA methods include all-ion fragmentation (AIF) (e.g., MS^All^, MS^E^) (Hu et al., 2016; Mun et al., 2019), where all precursors are fragmented. However, a critical drawback of the DDA mode is its limited MS^2^ spectral coverage, leaving a significant number of metabolic features without MS^2^ spectra for metabolite annotation, while the MS^2^ spectral quality of DIA is inferior to that of DDA, and the processing time of DIA data is much longer than that of DDA data. In this work, we proposed a novel strategy of DDA-assisted DIA to illustrate the chemicals in *P. chinense* (Figure 2). First, we used both DDA and DIA modes to acquire the mass spectra of *P. chinense*, which were converted into peak tables using MS-DIAL software. Then, the GNPS molecular network was constructed based on the peak table from the DDA mode, and more than three nodes and three edges molecular in GNPS were selected. After that, the selected molecules in the DDA GNPS network were inserted into the DIA peak table, which constructed a new peak table, and the DDA-assisted DIA GNPS network was constructed based on this new peak table (Figure 2A). In the DDA-assisted DIA GNPS network, only ions related to both DDA (blue node) and DIA (green node) are finally selected for identification (Figure 2B). Throughout the above analysis, 1,094 features were successfully screened (Figure 2C).

### DDA-Assisted Data-Independent Acquisition for Improved MS^2^ Quality

**FIGURE 3:**
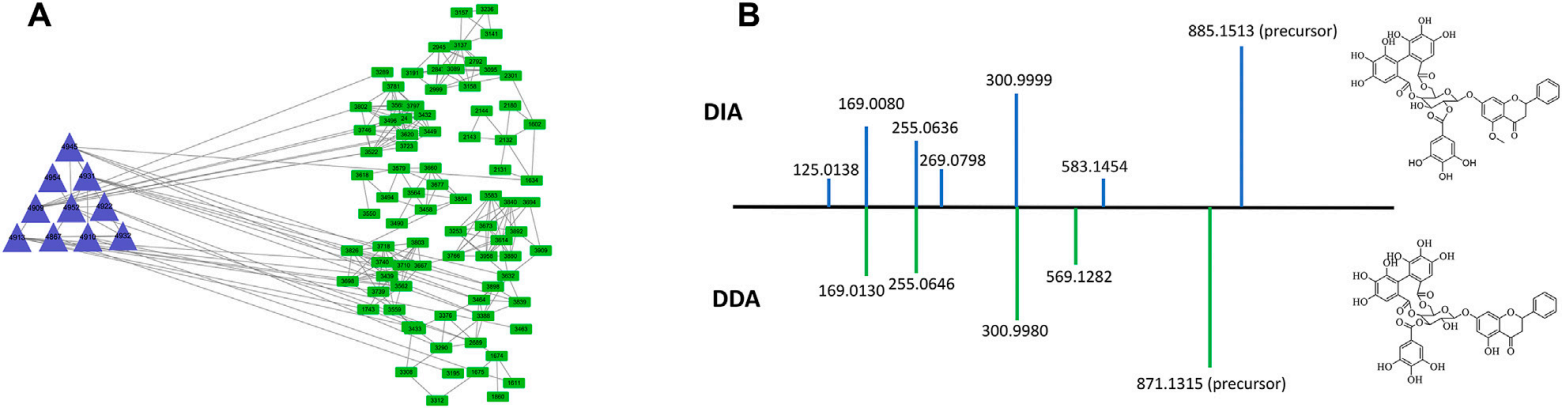
Representative example showing the effect of DDA-assisted DIA on the sensitive precursor ion. The MS features retrieved from DDA-assisted DIA **(A)**, with similar MS^2^ features of polyphenols in *P. chinense* **(B)**.

**FIGURE 4:**
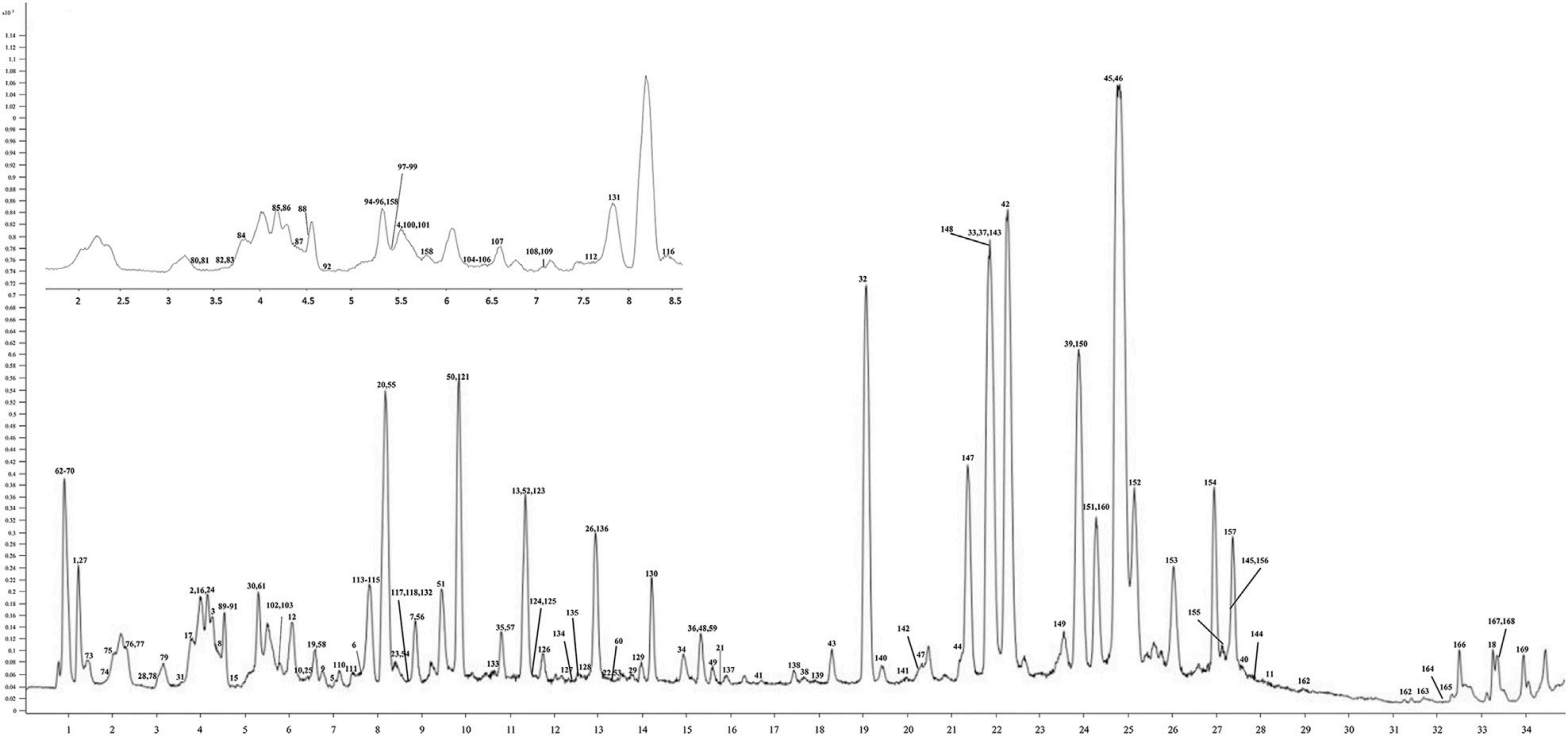
UHPLC-Q/TOF-MS chromatogram of the *P. chinense* extract used in the trial. The *X*-axis is the retention time, and the *Y*-axis is the absorbance unit.

To demonstrate the enhanced performance of DDA-assisted DIA, we took a specific cluster as an example. In Figure 3A, the purple triangle nodes were selected from the DDA molecular network, with characteristic fragments (300.9980, 255.0646, and 169.0130), which were typical MS^2^ features of polyphenols. In DDA-assisted DIA, the features (green quadrangles) were derived from the DIA mode, and those correlated with the DDA molecular network (purple triangle nodes) were considered as polyphenol candidate features (Figure 3A), which enhanced the screened features. Moreover, in the DIA mode, more MS^2^ features were detected compared to the DDA mode because of its chaotic fragmentation. DDA-assisted DIA could clearly identify the fragment ions in DIA (Figure 3B). In order to accurately demonstrate the chemicals in *P. chinense*, an in-house library, including 109 compounds (Supplementary Figure S1; Supplementary Table S1), was constructed based on previous chemical studies. After careful manual checking, 169 compounds were identified from *P. chinense*, including 121 in the DDA mode (Supplementary Table S2) (Figure 4). All the structure-deduced processes were characterized in Supplementary Tables S3, S4.

### Screening of Potential Bioactive Compounds and Candidate Targets in *Penthorum chinense* Pursh

**FIGURE 5:**
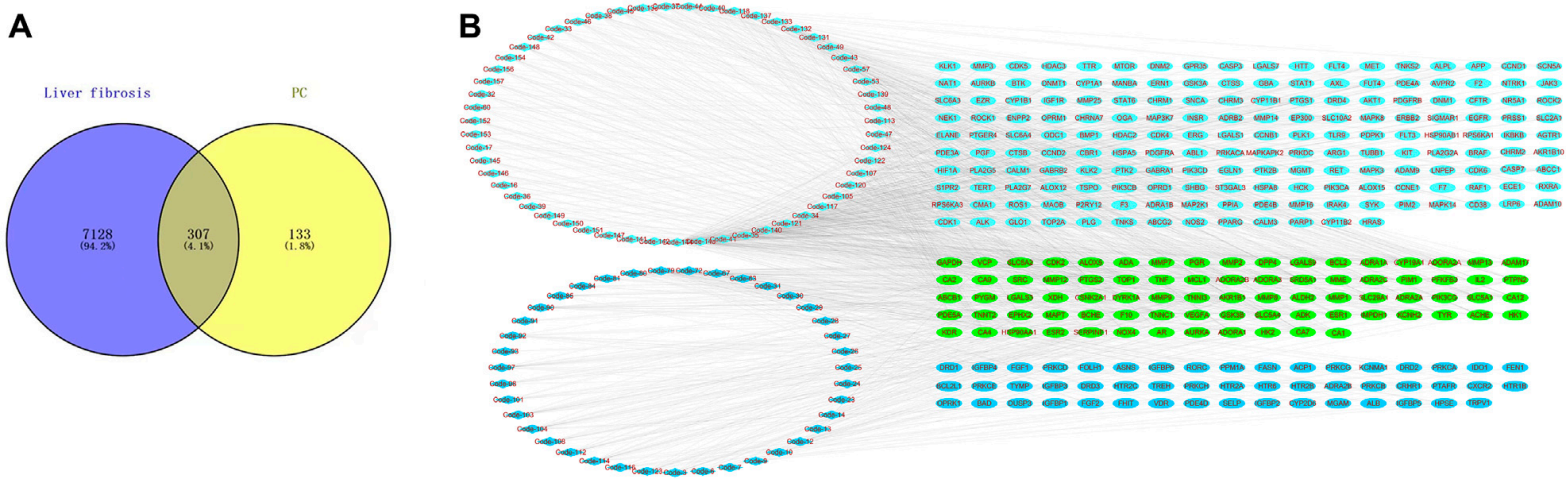
Network Pharmacology analysis of the *P. chinense* for liver fibrosis treatment. **(A)** Candidate target screening of *P. chinense* for liver fibrosis treatment. **(B)** Compound network of the *P. chinense* (light green represents flavonoids, and blue represents phenolic acids). The rectangle represents different compounds in *P. chinense*, and ellipse nodes represent active compounds.

Based on the TCMSP database (OB ≥ 30%) and the criterion of SwissTarget Prediction parameters, a total of 94 bioactive compounds were retrieved from *P. chinense*. Based on the screened compounds in *P. chinense*, 440 related targets were predicted (*p* < 0.05, Supplementary Table S5). For the disease “liver fibrosis,” a total number of 7,435 targets were also screened. Furthermore, a Venny assay showed that 307 targets overlapped between compound-related targets and disease-related targets (Figure 5A). In 94 bioactive compounds, 39 were assigned as phenols (blue diamond), which were targeted to 130 genes, while other 55 compounds were classified as flavonoids (light-green diamond), which were targeted to 255 genes, and 80 genes were shared by both types of compounds (Figure 5B).

### Target-Enriched Kyoto Encyclopedia of Genes and Genomes Pathway Analysis

**FIGURE 6:**
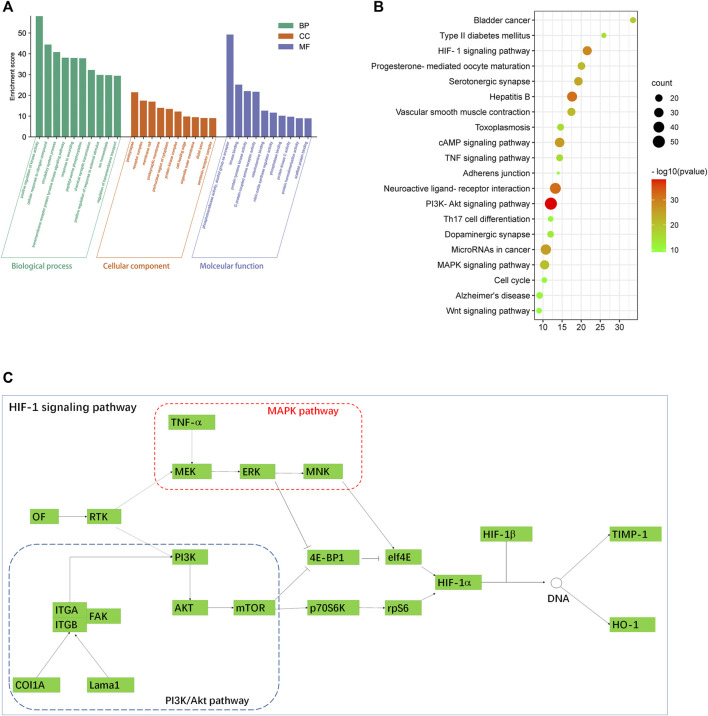
**(A)** GO enrichment analysis by the Metascape database. **(B)** Bubble chart of the target-enriched KEGG pathways. The bubble size represents the number of the targets in the enriched pathway terms, and the bubble color represents the pathway’s *p*-value. The HIF-1 pathway was founded to have a high number of targets enriched and a significant *p*-value. **(C)** HIF-1 pathway and its associated signaling pathways. Data were retrieved from the KEGG database (https://www.genome.jp/kegg/).

To explore the molecular mechanism of PC in liver fibrosis, GO enrichment analysis and KEGG enrichment were performed on the 440 candidate targets. A total of 2,329 biological processes, 147 cellular components, and 221 molecular functions were obtained by GO analysis, from which the top 10 terms were selected (*p* < 0.05, Supplementary Tables S7–S9) (Figure 6A). In addition, 198 pathways were found to be significantly associated with the input set of 269 targets through KEGG enrichment analysis, and the top 20 terms were selected (*p* < 0.05, Supplementary Table S6). From the bubble chart of the target enriched pathways, most of these pathways were enriched by multiple targets related to liver fibrosis (Figure 6B). The HIF-1 pathway analysis showed that multiple pathways interacted with the HIF-1 pathway, including the PI3K-Akt signaling pathway, MAPK signaling pathway, and vascular tone. It could be observed that some proteins, such as TNF-α, Timp1, and HO-1, were involved in the HIF-1 pathway (Figure 6C).

### *Penthorum chinense* Pursh Protects Against CCl_4_-Induced Liver Fibrosis

**FIGURE 7:**
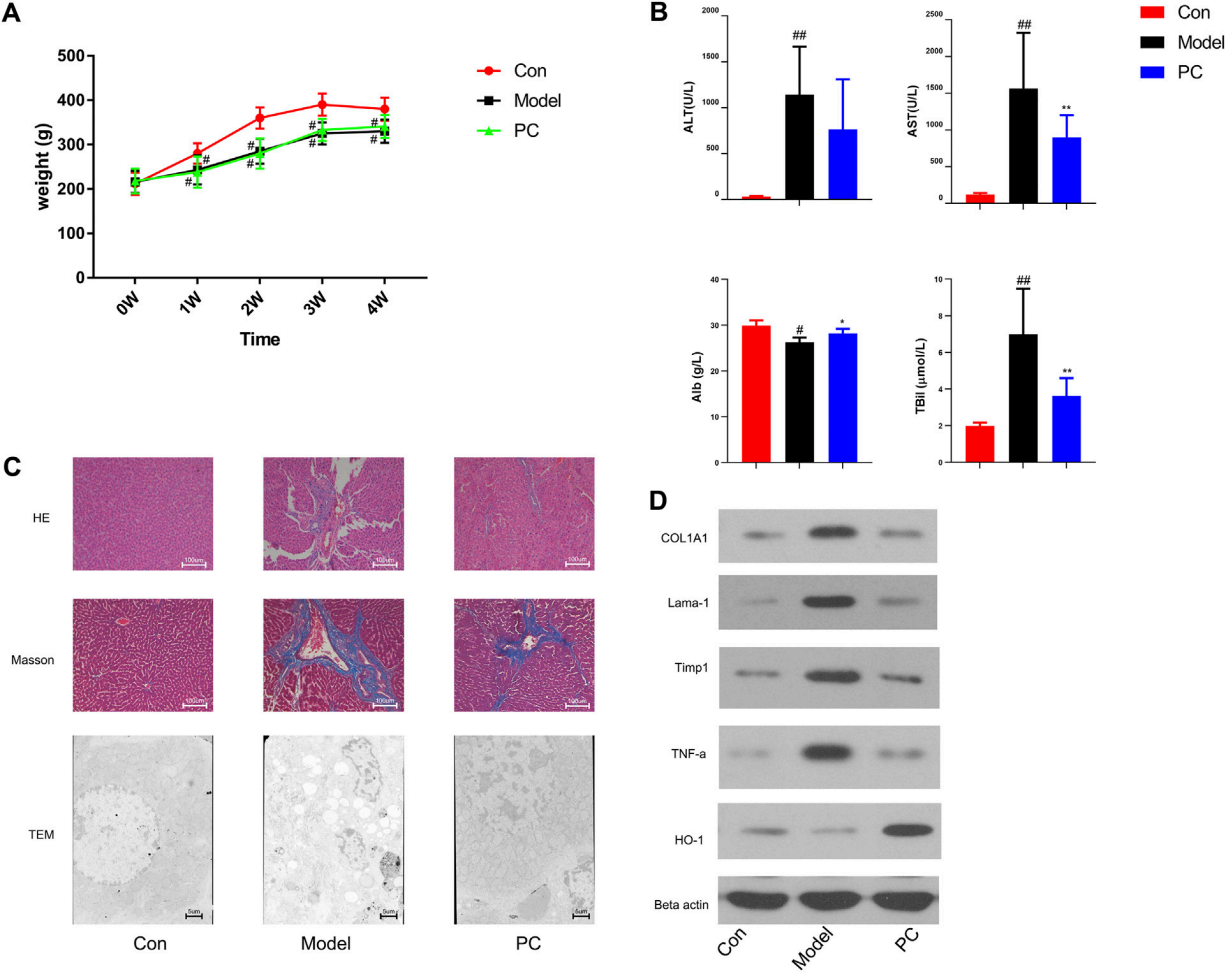
Evaluation of the therapeutic effect of *P. chinense* in CCl_4_-induced liver fibrosis rats **(A)** Body weight change (mean ± SD). **(B)** Levels of serum ATL, AST, Alb, and TBil in different groups. **(C)** HE staining (×100), Masson staining (×100), and TEM scanning (×500) of liver tissues in each group. **(D)** Expressions of COL1A, Lama-1, Timp1, TNF-α, and HO-1 in rat liver tissues were detected by Western blot. The data are presented as the means ± SDs of the results from three independent experiments and were analyzed by ANOVA. ^#^ *p* < 0.05, ^##^ *p* < 0.01 compared to the control group, and *p < 0.05, **p < 0.01 compared to the model group. PC, *P. chinense*.

In this study, the protective effect of *P. chinense* against CCl_4_-induced liver fibrosis in rats was examined. We found that severe liver injury was induced by CCl_4_ administration in rats. As shown in Figure 7A, CCl_4_ could significantly reduce the weight of rats over time, while the weight of rats in the PCP group did not change significantly compared with the CCl_4_ group. In addition, serum ATL and AST were detected to be considerably higher in the model group than in the control group (*p* < 0.01) (Figure 7B), indicating obvious liver function damage due to CCl_4_. Nevertheless, these changes were observed to be reversed by PC (*p* < 0.05 or *p* < 0.01) (Figure 7B). HE and Masson staining revealed that CCl_4_ resulted in collagen deposition, structural destruction, and inflammatory cell infiltration in liver lobules and portal areas. PC treatment was found to remarkably alleviate these histopathological lesions (Figure 7C). Then, the effect of PC on liver fibrosis was further examined by identifying the characteristic indicators of liver fibrosis, including COL1A1, Lama1, and Timp1. The higher quantities of COL1A1, Lama1, and Timp1 proteins were expressed in the model group, which were considerably subdued by PC administration (Figure 7D), signifying that PC reduced CCl_4_-induced liver fibrosis.

The network pharmacology analysis indicated that the *P. chinense* attenuates liver fibrosis potentially through the HIF-1 signaling pathway. As indicated in Figure 5B, the HIF-1 signaling pathway is involved in multiple genes, including inflammatory factors and hyperoxide-related genes. In this experiment, the protein expressions of TNF-α and HO-1 were significantly inhibited in the model group compared with the control group, and PC treatment significantly decreased gene expression levels compared to the model group.

## Discussion

Research on the chemical components is a prerequisite for elucidating the effective substances, mechanism of action, and clinical efficacy of TCM. However, the components of TCM are complex. Although traditional chemical isolation and identification could illustrate the chemicals of TCM, it is time-consuming and labor-intensive, and it is very difficult to identify trace components in TCM (WANGXiao-li, 2018; Li Dan et al., 2020). In recent years, LC-MS technology has been widely used in the analysis and research of TCM components, which avoids tedious preprocessing procedures and can obtain abundant information, such as compound retention time, molecular weight, and characteristic secondary structure fragments. Our research combines intelligent data recognition technology (DDA-assisted DIA data analysis) with network pharmacology to establish an efficient screening system for the effective components of *P. chinense*, which proves a strategy for discovering the efficacy of TCM.

In various efforts, an important research direction is to develop an MS data acquisition mode to improve the performance of metabolite detection, quantification, and annotation (Bruderer et al., 2015; Wahman et al., 2021). In the DIA mode, the MS^2^ spectrum is not generated specifically for any single metabolic ion but for a group of parent ions with a wide range of *m/z* values (Siegel et al., 2014; Reubsaet et al., 2019). Common DIA methods include AIF (e.g., MS^all^ and MS^E^), in which all precursors are fragmented (Siegel et al., 2014; Schrimpe-Rutledge et al., 2016). In the analysis of DIA metabolomics data, additional deconvolution integration is required to associate each fragment ion with its corresponding precursor metabolic characteristics (Guo and Huan, 2020). However, the previous comparison shows that the MS^2^ spectral quality of DIA is not as good as that of the DDA mode, in which the MS switches between collecting the MS^1^ and MS^2^ spectra so that both the quantitative information and the fragmentation information are recorded (Li et al., 2021). Moreover, the processing time of DIA data is much longer than that of DDA data. In order to further improve MS data acquisition to generate high-quality metabolomics data, we seek to create a new data acquisition workflow integrating the advantages of DDA and DIA modes. Previous studies reported 27 compounds and 50 compounds based on DIA and DDA methods, respectively (Guo et al., 2015; Yin et al., 2020). In this research, we developed a new method for combining DDA and DIA data to obtain high-quality MS^1^ and MS^2^ of *P. chinense*, and 169 compounds were identified from *P. chinense* after manual checking. Among 169 identified compounds, 38 were confirmed with reference standards, while 131 were tentatively identified based on their accurate mass weight, fragmentations, and comparison with previous reports. Macrocyclic polyphenols, with an HHDP group, were a very interesting class of compounds in *P. chinense*, which exhibited various biological activities (Huang et al., 2014; Huang et al., 2015b). In this study, three macrocyclic polyphenols were used as references, and 15 other macrocyclic polyphenols were identified through DDA-assisted DIA methods.

Liver fibrosis is a nonhealing response to wounds under persistent injury factors characterized by excessive accumulation of the extracellular matrix (ECM) (Hernandez-Gea and Friedman, 2011). CCl_4_ belongs to the class of hepatotoxins and functions after metabolic activation. It is generally believed that CCl_4_ normally enters hepatocytes and forms free radicals to cause peroxidation, resulting in liver structural damage and liver function impairment (Li et al., 2017; Xu et al., 2019). The increase of CCl_4_-induced reactive oxygen species may cause tissue damage through lipid peroxidation, increase the expression of the tissue inhibitor of metalloproteinase-1 (TIMP-1), and cause liver fibrosis due to the accumulation of collagen in the liver (Hafez et al., 2015). *Penthorum chinense* has been long considered to have an anti-liver fibrosis effect both *in vivo* and *in vitro*. Previous reports used both alcoholic and CCl_4_-induced liver fibrosis to confirm the anti-liver fibrosis of *P. chinense*. In the CCl_4_ model, the *P. chinense* extract (Gansu Granules) significantly alleviated liver fibrosis via inhibiting α-SMA and collagen I and III expressions, which were very similar to our research (Wang et al., 2020). In this study, the CCl_4_-treated group showed significant collagen accumulation by Masson analysis, and the *P. chinense* exhibited a protective effect against collagen deposition. In addition, *P. chinense* could attenuate liver pathological change through enhancing the liver capacity of antioxidant and anti-inflammatory properties, while *P. chinense* has been confirmed to have anti-liver fibrosis via the HIF-1 pathway in the KEGG pathway analysis. HIF-1 expressed in hepatocytes promotes adaptive mechanisms, including increased glucose transport and HO-1, which may compensate for the interruption of mitochondrial functions and the increase of ROS (Wang et al., 2021b). Early studies have shown that HIF-1 could induce HO-1 in hepatocytes for protecting the liver from fibrosis promoted by cholate supplementation. Because HO-1 has been proved to be associated with the inhibition of hepatic stellate cell activation and reverses the progression of fibrosis (Han et al., 2019). It has been reported that CCl_4_ could activate Kupffer cells that produce pro-inflammatory cytokines through upregulating TNF-α, monocyte chemoattractant protein-1, macrophage inflammatory protein-2, IL -1β, IL-6, profibrotic cytokine TGF-21, and nuclear factor mB p65 protein expression in the CCl_4_-induced liver fibrosis model (Unsal et al., 2021). In our research, *P. chinense* significantly promoted HO-1 expression in rat liver tissues. Moreover, inflammation factors are involved in the HIF-1 pathway according to KEGG pathway analysis, including NF-κB and MAPK pathways, and the TNF-α expression was significantly upregulated in the CCl_4_-treated group, while *P. chinense* could reverse this trend. Thus, *P. chinense* attenuated liver fibrosis *via* HIF-1 pathway regulation.

Within the bioactive compounds screened from *P. chinense*, 94 out of 169 were identified with liver fibrosis progress. Through the compound–target–network analysis, flavonoids and phenols showed a high correlation with liver fibrosis genes. In the compound–target–network analysis (Supplementary Table S5), catechin-3-O-gallate was related to 37 targets, which were involved in the HIF-1 signal pathway, PI3K-Akt pathway, and ECM–receptor interaction. Coincidentally, a previous study also revealed that epicatechin-3-O-gallate displayed a significant anti-hepatic fibrosis effect by suppressing collagen production and collagenase activity in hepatic stellate cells, which was similar to our study (Nakamuta et al., 2005). Another flavonoid, quercetin, was related to 21 targets, which were involved in the IL-17 signaling pathway. A previous study revealed that IL-17 directly stimulated collagen expression in Hepatic stellate cells (HSCs) *via* the Stat3 signaling pathway (Meng et al., 2012). Overall, in our research, multiple genes were attributed to the HIF-1 signaling pathway, which interacted with other pathways, including the PI3K-Akt signaling pathway, MAPK signaling pathway, and vascular tone. It could be observed that some proteins, such as TNF-α, Timp1, and HO-1, were involved in the HIF-1 pathway. In addition, in our *in vivo* experiment, *P. chinense* could significantly suppress the protein expression of TNF-α and reversed the expression of HO-1 after CCl_4_ treatment.

In summary, we developed a DDA-assisted DIA to enhance both MS^1^ acquisition and MS^2^ quality, which may benefit compound identification in LC-MS analysis. Through the DDA-assisted DIA analysis, 169 compounds were identified in *P. chinense*. In order to figure out the correlation between the compounds and liver fibrosis, network pharmacology analysis revealed that 94 compounds were related to liver fibrosis. According to the KEGG pathway analysis, *P. chinense* attenuated liver fibrosis through regulation of the HIF-1 signaling pathway, which was confirmed by the *in vivo* experiment. This work provides a new perspective to screen and investigate the constituents of *P. chinens*e. Combined with network pharmacology, this research might serve as an automatic template for discovering the complex effective constituents of TCM. Above all, there were several defects in this research: 1) the animal dose should be optimized and 2) the most relevant chemicals of liver fibrosis in *P. chinense* should be chosen for activity verification.

## Data Availability

The original contributions presented in the study are included in the article/Supplementary Materials, further inquiries can be directed to the corresponding author.
