# Corrigendum: Integrated chemical interpretation and network pharmacology analysis to reveal the anti-liver fibrosis effect of *Penthorum chinense*

**DOI:** 10.3389/fphar.2022.986072

**Published:** 2022-08-09

**Authors:** Zenan Du, Doudou Huang, Pengjie Shi, Zhiying Dong, Xiujuan Wang, Mengshuang Li, Wansheng Chen, Feng Zhang, Lianna Sun

**Affiliations:** ^1^ School of Pharmacy, Shanghai University of Traditional Chinese Medicine (SHUTCM), Shanghai, China; ^2^ Institute of Chinese Materia Madica, Shanghai University of Traditional Chinese Medicine, Shanghai, China; ^3^ Department of Pharmacy, Changzheng Hospital, Second Military Medical University, Shanghai, China

**Keywords:** DDA-assisted DIA, network pharmacology, action of mechanism, *Penthorum chinense*, liver fibrosis

In the original article, there was a mistake in Figure 7 as published. “After carefully checked, the band of Timp1 was covered by TNF-α in Figure 7D during figure software processing which caused by duplication of TNF-α. In addition, the units of Alb and TBil should be g/L and µmol/L, respectively, after checking the raw data.” The corrected Figure 7 is as follows.

**FIGURE 7:**
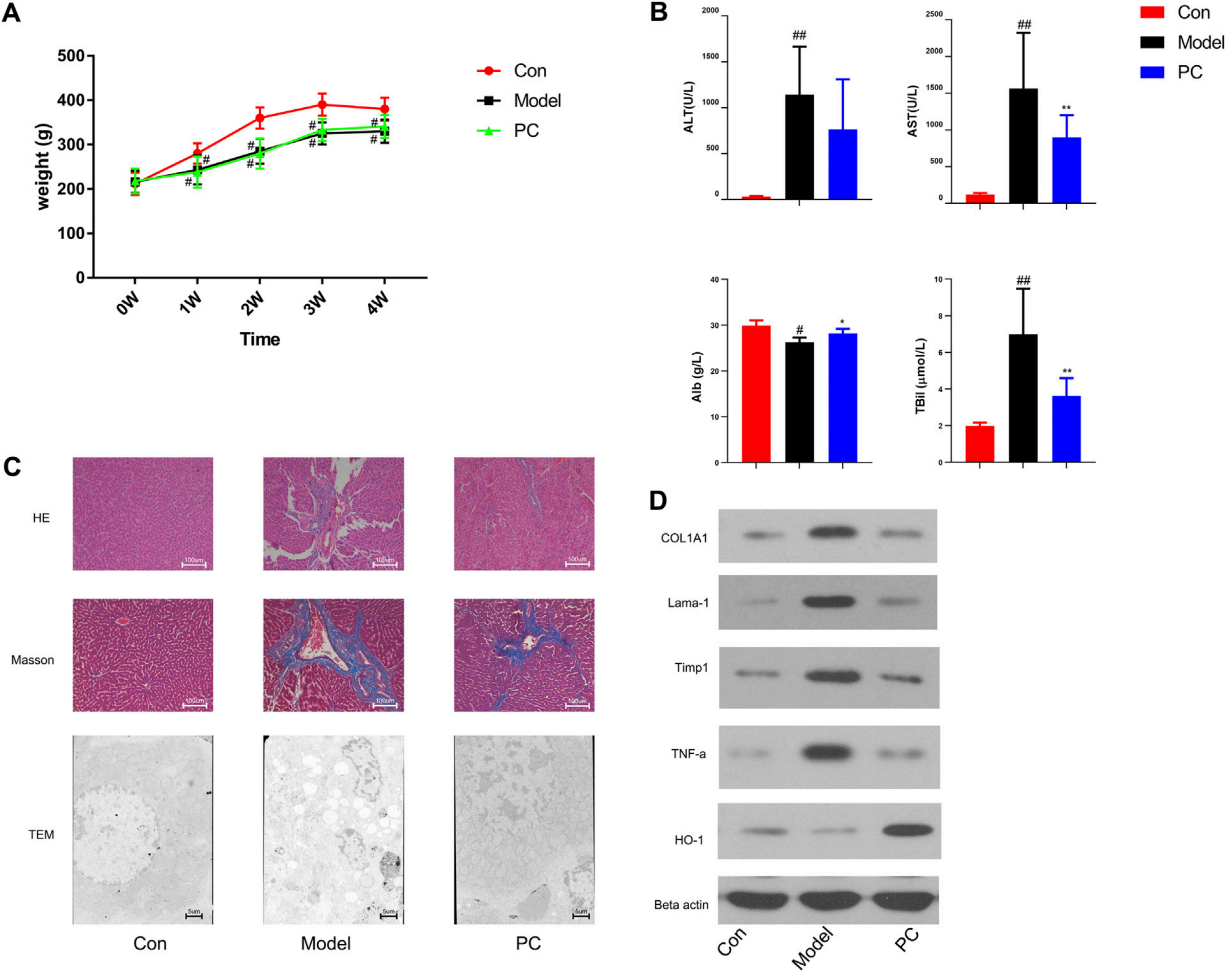
Evaluation of the therapeutic effect of *P. chinense* in CCl_4_-induced liver fibrosis rats. **(A)** Body weight change (mean ± SD). **(B)** Levels of serum ATL, AST, Alb, and TBil in different groups. **(C)** HE staining (×100), Masson staining (×100), and TEM scanning (×500) of liver tissues in each group. **(D)** Expressions of COL1A, Lama-1, Timp1, TNF-α, and HO-1 in rat liver tissues were detected by Western blot. The data are presented as the means ± SDs of the results from three independent experiments and were analyzed by ANOVA. ^#^ *p* < 0.05, ^##^ *p* < 0.01 compared to the control group, and ∗*p* < 0.05, ∗∗*p* < 0.01 compared to the model group. PC, *P. chinense*.

The authors apologize for this error and state that this does not change the scientific conclusions of the article in any way. The original article has been updated.

