# Supplementary material for: Integrated Chemical Interpretation and Network Pharmacology Analysis to Reveal the Anti-Liver Fibrosis Effect of Penthorum chinense
Source: Front Pharmacol. 2022 Jun 2;13:788388. doi: 10.3389/fphar.2022.788388 (PMC9201443; doi:10.3389/fphar.2022.788388)
Supplement: Supplementary file 6 [file Table1.doc]

Additional file table S1. In-house library of *P. chinese*.

| **Code** | **Name** | **Formula** |
| --- | --- | --- |
| pc-1 | gallic acid | C_7_H_6_O_5_ |
| pc-2 | methyl gallate | C_8_H_8_O_5_ |
| pc-3 | 1-O-galloyl-4,6-(R)-HHDP-β-D-glucose | C_27_H_22_O_18_ |
| pc-4 | strictinin | C_27_H_22_O_18_ |
| pc-5 | ethyl gallate | C_9_H_10_O_5_ |
| pc-6 | ellagic acid | C_14_H_6_O_8_ |
| pc-7 | ethyl brevifolincarboxylate | C_15_H_12_O_8_ |
| pc-8 | brevifolin-carboxylic acid | C_13_H_8_O_8_ |
| pc-9 | methyl brevifolincarboxylate | C_14_H_10_O_8_ |
| pc-10 | brevifolin | C_12_H_8_O_6_ |
| pc-11 | 9,9′-O-Diferuloyl-(-)secoisolariciresinol | C_40_H_42_O_12_ |
| pc-12 | 2′,4′,6′-trihydroxyacetophenone 4′-O-β-glucoside | C_14_H_18_O_9_ |
| pc-13 | 2,4,6-trihydroxybenzoic acid | C_7_H_6_O_5_ |
| pc-14 | 2′,4′,6′-trihydroxyacetophenone 4′-O-[4,6-(R)-HHDP]-β-glucoside | C_28_H_24_O_17_ |
| pc-15 | 2′,4′,6′-trihydroxyacetophenone 4′-O-[4,6-(S)-HHDP]-β-glucoside | C_28_H_24_O_17_ |
| pc-16 | bergeninum | C_14_H_16_O_9_ |
| pc-17 | 4-O-galloylbergenin | C_21_H_20_O_13_ |
| pc-18 | 11-O-galloylbergenin | C_21_H_20_O_13_ |
| pc-19 | protocatechuic acid | C_7_H_6_O_4_ |
| pc-20 | vanillic acid | C_8_H_8_O_4_ |
| pc-21 | epicatechin | C_15_H_14_O_6_ |
| pc-22 | catechin | C_15_H_14_O_6_ |
| pc-23 | ferruginol | C_20_H_30_O |
| pc-24 | β-1,4,6-tri-O-galloyl-D-glucose | C_27_H_24_O_18_ |
| pc-25 | (-)-epicatechin-3-O-gallate | C_22_H_18_O_10_ |
| pc-26 | (-)-(7R,8S)-4,7,9,3',9'-pentahydroxy-3-methoxy-8-4'-oxyneolignan-3'-O-β-D-glucopyranoside | C_25_H_34_O_12_ |
| pc-27 | penthorumin A | C_25_H_28_O_11_ |
| pc-28 | penthorumin B | C_25_H_28_O_11_ |
| pc-29 | penthorumin C | C_19_H_22_O_11_ |
| pc-30 | penthorumin D | C_26_H_24_O_17_ |
| pc-31 | penthorumnin A | C_27_H_24_O_19_ |
| pc-32 | penthorumnin B | C_12_H_10_O_8_ |
| pc-33 | penthorumnin C | C_27_H_24_O_17_ |
| pc-34 | penthorumnin D | C_28_H_24_O_18_ |
| pc-35 | balanophotannin F | C_28_H_24_O_17_ |
| pc-36 | phyllanemblinin F | C_27_H_26_O_20_ |
| pc-37 | chebulic acid | C_14_H_12_O_11_ |
| pc-38 | helicin | C_13_H_16_O_7_ |
| pc-39 | 1,3,5-trihydroxybenzene 1-O-[4,6-(S)-HHDP]-β-D-glucoside | C_26_H_22_O_16_ |
| pc-40 | (E)-phenylpropene-3-methoxyphenyl-[6′′-O-galloy]-4-O-β-D-glucopyranoside | C_23_H_26_O_11_ |
| pc-41 | 2,6-dihydroxyacetophenone-5-(2′-methylene-2(5H)-furanone)-4-O-β-D-glucopyranoside | C_19_H_22_O_11_ |
| pc-42 | 1-O-sinapoyl-β-D-glucopyranoside | C_17_H_22_O_10_ |
| pc-43 | ferulic acid glucopyranoside | C_16_H_20_O_9_ |
| pc-44 | scopoletin | C_10_H_8_O_4_ |
| pc-45 | pinocembrin-7-O-[4′′,6′′-(S)-hexahydroxydiphenoyl]-β-D-glucoside | C_35_H_28_O_17_ |
| pc-46 | pinocembrin-7-O-[3′′-O-galloyl-4′′,6′′-(S)-hexahydroxydiphenoyl]-β-D-glucoside | C_42_H_32_O_21_ |
| pc-47 | pinocembrin-7-O-β-D-glucopyranoside | C_21_H_22_O_9_ |
| pc-48 | alpinetin-7-O-β-D-glucopyranoside | C_22_H_24_O_9_ |
| pc-49 | naringenin | C_15_H_12_O_5_ |
| pc-50 | pinocembirn | C_15_H_12_O_4_ |
| pc-51 | pinocembrin-7-O-[3′′-O-galloyl]-β-D-glucose | C_28_H_26_O_13_ |
| pc-52 | pinocembrin-7-O-[2′′-O-galloyl-4′′,6′′-hexahydroxydiphenoyl]-β-D-glucose | C_42_H_32_O_21_ |
| pc-53 | pinostrobin | C_16_H_14_O_4_ |
| pc-54 | naringenin-7-O-glucoside | C_21_H_22_O_10_ |
| pc-55 | (E)-3-phenyl-1-(2,4,6-trihydroxyphenyl)prop-2-en-1-one | C_15_H_12_O_4_ |
| pc-56 | pinostrobin chalcone | C_16_H_14_O_4_ |
| pc-57 | thonningianin B | C_35_H_30_O_17_ |
| pc-58 | 2′,4′,6′-trihydroydihychalcone-4′-β-D-glucoside | C_21_H_24_O_9_ |
| pc-59 | 2′,6′-dihydroydihychalcone-4′-O-[3′′-O-galloy]-β-D-glucoside | C_28_H_28_O_13_ |
| pc-60 | 2′,6′-dihydroxydihydrochalcone-4′-O-[2′′-O-galloyl-4′′,6′′-hexahydroxydiphenoyl]-β-D-glucopyranoside | C_42_H_34_O_21_ |
| pc-61 | thonningianin A | C_42_H_34_O_21_ |
| pc-62 | 6′-hydroxy-2′-methoxy-dihydrochalcone-4′-O-β-D-glucopyranoside | C_22_H_26_O_9_ |
| pc-63 | 4-deoxy-phlorizin | C_21_H_24_O_9_ |
| pc-64 | kaempferol | C_15_H_10_O_6_ |
| pc-65 | quercitrin | C_21_H_20_O_11_ |
| pc-66 | quercetin-3-O-α-L-arabinoside | C_20_H_18_O_11_ |
| pc-67 | kaempferol-3-O-α-L-rhamnopyranoside | C_21_H_20_O_10_ |
| pc-68 | quercetin | C_15_H_10_O_7_ |
| pc-69 | isoqueritrin | C_21_H_20_O_12_ |
| pc-70 | rutin | C_27_H_30_O_16_ |
| pc-71 | quercetin-3-O-β-D-xyloside | C_20_H_18_O_11_ |
| pc-72 | quercetin-3′-O-α-L-rhamnoside | C_21_H_20_O_11_ |
| pc-73 | kaempferol-3-O-arabinoside | C_20_H_18_O_10_ |
| pc-74 | kaempferol-3-O-rutinoside | C_28_H_32_O_14_ |
| pc-75 | quercetin-3-O-β-D-glucopyranosyl-(1→2)-β-D-glucopyranoside | C_27_H_30_O_17_ |
| pc-76 | quercetin-3-O-sambubioside | C_25_H_26_O_17_ |
| pc-77 | spiraeoside | C_21_H_20_O_12_ |
| pc-78 | quercetin di-O-glycoside | C_27_H_30_O_17_ |
| pc-79 | apigenin | C_15_H_10_O_5_ |
| pc-80 | chrysin | C_21_H_20_O_9_ |
| pc-81 | apigenin-7-O-β-D-apiofuranosyl(1→2)-β-D-glucopyranoside | C_26_H_28_O_14_ |
| pc-82 | luteolin | C_15_H_10_O_6_ |
| pc-83 | scutellarin-7-O-α-L-rhamnoside | C_21_H_20_O_10_ |
| pc-84 | daidzein | C_15_H_10_O_4_ |
| pc-85 | mangiferin | C_19_H_18_O_11_ |
| pc-86 | hispidulin-7-(6-E-p-coumaroyl-β-D-glucopyranoside) | C_31_H_28_O_13_ |
| pc-87 | penchinoneA | C_19_H_18_O_6_ |
| pc-88 | penchinoneB | C_19_H_18_O_6_ |
| pc-89 | penchinoneC | C_19_H_18_O_5_ |
| pc-90 | penchinoneD | C_19_H_18_O_5_ |
| pc-91 | penthorin A | C_19_H_18_O_5_ |
| pc-92 | penthorin B | C_19_H_18_O_5_ |
| pc-93 | (7′E)-2′,4,8-trihydroxy-3-methoxy-2,4′-epoxy-8,5′-neolign-7′-en-7-one | C_19_H_18_O_6_ |
| pc-94 | (7′Z,8S)-4,8-dihydroxy-3-methoxy-2,4′-epoxy-8,5′-neolign-7′-en-7-one-2′-O-β-D-glucopyranose | C_25_H_28_O_11_ |
| pc-95 | (7′Z,8S)-3,8-dihydroxy-4-methoxy-2,4′-epoxy-8,5′-neolign-7′-en-7-one-2′-O-β-D-glucopyranose | C_25_H_28_O_11_ |
| pc-96 | (+)-syringaresinol | C_22_H_26_O_8_ |
| pc-97 | (+)-medioresinol | C_21_H_24_O_7_ |
| pc-98 | (+)-pinoresinol | C_20_H_22_O_6_ |
| pc-99 | (+)-episyringaresinol | C_22_H_26_O_8_ |
| pc-100 | (+)-epipinoresinol | C_20_H_22_O_6_ |
| pc-101 | 4,4'-dimethoxy-3'-hydroxy-7,9',7',9-diepoxylignan-3-O-β-D-glucopyranoside | C_26_H_32_O_11_ |
| pc-102 | (-)-syringaresinol | C_22_H_26_O_8_ |
| pc-103 | β-sitosterol | C_29_H_50_O |
| pc-104 | β-daucosterol | C_35_H_60_O_6_ |
| pc-105 | 2-hydroxyl-28-O-[α-L-rhamnopyranosyl-(1→2)-β-D-glucopyranoside]-ursolic acid | C_42_H_68_O_11_ |
| pc-106 | ursolic acid | C_30_H_48_O_3_ |
| pc-107 | lupeol | C_30_H_50_O |
| pc-108 | betulinic acid | C_30_H_48_O_3_ |
| pc-109 | 2β,3β,23-trihydroxy-urs-12-ene-28-olic acid | C_29_H_46_O_5_ |
