# Supplementary material for: Integrated Chemical Interpretation and Network Pharmacology Analysis to Reveal the Anti-Liver Fibrosis Effect of Penthorum chinense
Source: Front Pharmacol. 2022 Jun 2;13:788388. doi: 10.3389/fphar.2022.788388 (PMC9201443; doi:10.3389/fphar.2022.788388)
Supplement: Supplementary file 10 [file Table2.doc]

**Supplementary file table S2. Characterization of *P. chinese*.**

| **Code** | **Name** | **Formula** | **MS^1^** | **RT (min)** | **MS^2^** | **Error (ppm)** |
| --- | --- | --- | --- | --- | --- | --- |
| 1* | gallic acid | C_7_H_6_O_5_ | 169.0142 | 1.470 | 125.02 | 0.5916 |
| 2* | methyl gallate | C_8_H_8_O_5_ | 183.0289 | 4.067 | 169.01,124.01 | 4.8735 |
| 3* | 1-O-galloyl-4,6-(R)-HHDP-β-D-glucose | C_27_H_22_O_18_ | 633.0723 | 4.283 | 463.04,300.99,169.01 | 1.6361 |
| 4* | strictinin | C_27_H_22_O_18_ | 633.0723 | 5.540 | 463.04,300.99,169.01 | -0.0987 |
| 5* | ethyl gallate | C_9_H_10_O_5_ | 197.0460 | 7.040 | 367.06,246.02,183.02,168.00,124.01 | 2.5374 |
| 6* | ellagic acid | C_14_H_6_O_8_ | 300.9981 | 7.709 | 229.01 | -0.3629 |
| 7* | ethyl brevifolincarboxylate | C_15_H_12_O_8_ | 319.0450 | 8.933 | 273.00,245.00,217.00,189.01 | -2.8209 |
| 8* | brevifolin-carboxylic acid | C_13_H_8_O_8_ | 291.0137 | 4.365 | 247.02,219.02,191.03 | 3.2217 |
| 9* | methyl brevifolincarboxylate | C_14_H_10_O_8_ | 305.0296 | 6.797 | 611.06,273.00,245.00,217.01,189.01 | 1.9305 |
| 10* | brevifolin | C_12_H_8_O_6_ | 247.0241 | 6.185 | 219.03,191.03,167.03,123.04 | 2.8686 |
| 11* | 9,9′-O-Diferuloyl-(-)secoisolariciresinol | C_40_H_42_O_12_ | 713.2595 | 27.836 | 537.20,519.20,383.14,193.05,175.03 | 1.0504 |
| 12* | 2′,4′,6′-trihydroxyacetophenone 4′-O-β-glucoside | C_14_H_18_O_9_ | 329.0874 | 6.097 | 659.18,392.08,365.06,191.03,167.03,123.04 | 1.2290 |
| 13* | 2′,4′,6′-trihydroxyacetophenone 4′-O-[4,6-(R)-HHDP]-β-glucoside | C_28_H_24_O_17_ | 631.0941 | 11.403 | 300.99,167.03 | -0.2011 |
| 14* | 2′,4′,6′-trihydroxyacetophenone 4′-O-[4,6-(S)-HHDP]-β-glucoside | C_28_H_24_O_17_ | 631.0941 | 11.403 | 300.99,167.03 | -0.2011 |
| 15* | vanillic acid | C_8_H_8_O_4_ | 167.0356 | 4.756 | 152.01,108.02 | 3.5920 |
| 16* | epicatechin | C_15_H_14_O_6_ | 289.0711 | 4.021 | 352.06,325.04,179.03,151.03 | 1.9364 |
| 17* | catechin | C_15_H_14_O_6_ | 289.0707 | 3.881 | 352.06,325.04,179.03,151.03 | 3.6600 |
| 18* | ferruginol | C_20_H_30_O | 285.2225 | 33.273 | 241.21 | 0.3506 |
| 19* | β-1,4,6-tri-O-galloyl-D-glucose | C_27_H_24_O_18_ | 635.0883 | 6.601 | 465.06,295.04,169.01 | 1.0808 |
| 20* | (-)-epicatechin-3-O-gallate | C_22_H_18_O_10_ | 441.0814 | 8.213 | 504.07,477.05,289.07,245.08,169.07 | 2.7603 |
| 21* | penthorumin A | C_25_H_28_O_11_ | 503.1558 | 15.737 | 341.10,323.09,308.06,299.09,201.05,139.04,124.01 | 0.1987 |
| 22* | penthorumin B | C_25_H_28_O_11_ | 503.1555 | 13.431 | 566.15,341.10,323.09,308.06,299.09,201.05,139.04,124.01 | -0.5962 |
| 23* | penthorumin D | C_26_H_24_O_17_ | 607.0943 | 8.397 | 289.07,273.00,169.01 | 2.2577 |
| 24* | penthorumnin A | C_27_H_24_O_19_ | 651.0833 | 4.182 | 499.07,471.07,351.04,169.01 | 0.9233 |
| 25* | penthorumnin C | C_27_H_24_O_17_ | 619.0933 | 6.200 | 365.05,300.99 | 1.0851 |
| 26* | penthorumnin D | C_28_H_24_O_18_ | 647.0885 | 12.966 | 298.98 | 0.5979 |
| 27* | chebulic acid | C_14_H_12_O_11_ | 355.0312 | 1.223 | 249.04,205.05,193.01,163.04,151.03 | 1.4083 |
| 28* | 1,3,5-trihydroxybenzene 1-O-[4,6-(S)-HHDP]-β-D-glucoside | C_26_H_22_O_16_ | 589.0836 | 2.733 | 300.99,123.00 | -0.1600 |
| 29* | (E)-phenylpropene-3-methoxyphenyl-[6′′-O-galloy]-4-O-β-D-glucopyranoside | C_23_H_26_O_11_ | 477.1402 | 13.771 | 313.05,169.01,163.07,125.02 | 0.4191 |
| 30* | 1-O-sinapoyl-β-D-glucopyranoside | C_17_H_22_O_10_ | 633.0733 | 5.396 | 448.10,421.09,205.05 | 1.8660 |
| 31* | ferulic acid glucopyranoside | C_16_H_20_O_9_ | 355.1044 | 5.196 | 175.03 | -2.9322 |
| 32* | pinocembrin-7-O-[4′′,6′′-(S)-hexahydroxydiphenoyl]-β-D-glucoside | C_35_H_28_O_17_ | 719.1253 | 19.101 | 300.99,257.08 | -0.0375 |
| 33* | pinocembrin-7-O-[3′′-O-galloyl-4′′,6′′-(S)-hexahydroxydiphenoyl]-β-D-glucoside | C_42_H_32_O_21_ | 871.1364 | 21.902 | 569.12,435.05,300.99,255.06,169.01 | -0.0784 |
| 34* | pinocembrin-7-O-β-D-glucopyranoside | C_21_H_22_O_9_ | 417.1179 | 14.971 | 480.11,463.12,255.06,151.00 | 2.8838 |
| 35* | alpinetin-7-O-β-D-glucopyranoside | C_22_H_24_O_9_ | 431.1353 | 10.839 | 494.13,477.14,269.08,165.01 | -1.4905 |
| 36* | naringenin | C_15_H_12_O_5_ | 271.0603 | 15.395 | 151.00,125.02 | 3.297 |
| 37* | pinocembirn | C_15_H_12_O_4_ | 255.0672 | 22.118 | 151.00,107.01 | 3.5284 |
| 38* | pinocembrin-7-O-[3′′-O-galloyl]-β-D-glucose | C_28_H_26_O_13_ | 569.1303 | 17.694 | 313.05,255.06,169.01 | -0.4132 |
| 39* | pinocembrin-7-O-[2′′-O-galloyl-4′′,6′′-(S)-hexahydroxydiphenoyl]-β-D-glucoside | C_42_H_32_O_21_ | 871.1360 | 23.934 | 569.12,435.05,300.99,255.06,169.01 | 0.3802 |
| 40* | pinostrobin | C_16_H_14_O_4_ | 269.0807 | 27.672 | 165.02 | 4.1931 |
| 41* | pinostrobin chalcone | C_16_H_14_O_4_ | 269.0776 | 16.570 | 255.06,167.03,165.01,125.02 | -1.3600 |
| 42* | thonningianin B | C_35_H_30_O_17_ | 721.1404 | 22.315 | 463.04,300.99,257.08 | 0.8627 |
| 43* | 2′,4′,6′-trihydroydihychalcone-4′-β-D-glucoside | C_21_H_24_O_9_ | 419.1344 | 18.321 | 482.13,455.11,257.08,213.09,125.02 | 0.6091 |
| 44* | 2′,6′-dihydroydihychalcone-4′-O-[3′′-O-galloy]-β-D-glucoside | C_28_H_28_O_13_ | 571.1442 | 21.349 | 634.13,313.05,257.08.169.01.125.02 | 2.6470 |
| 45* | 2′,6′-dihydroxydihydrochalcone-4′-O-[2′′-O-galloyl-4′′,6′′-hexahydroxydiphenoyl]-β-D-glucopyranoside | C_42_H_34_O_21_ | 873.1513 | 24.879 | 569.12,435.05,300.99,255.06,169.01 | 0.7798 |
| 46* | thonningianin A | C_42_H_34_O_21_ | 873.1508 | 24.778 | 571.14,435.05,300.99,257.08,169.01 | 1.3518 |
| 47* | 6′-hydroxy-2′-methoxy-dihydrochalcone-4′-O-β-D-glucopyranoside | C_22_H_26_O_9_ | 433.1499 | 20.409 | 496.14,271.09,169.01,152.01,124.01 | 1.1655 |
| 48* | 4-deoxy-phlorizin | C_21_H_24_O_9_ | 419.1341 | 15.358 | 839.27,482.12,455.11,257.08,213.09,125.02 | 1.3231 |
| 49* | kaempferol | C_15_H_10_O_6_ | 285.0393 | 15.627 | 229.05,151.00,125.02 | 4.0609 |
| 50* | quercitrin | C_21_H_20_O_11_ | 447.0923 | 9.873 | 895.19,510.08,483.06,300.02,255.03,151.00 | 2.1982 |
| 51* | quercetin-3-O-α-L-arabinoside | C_20_H_18_O_11_ | 433.0761 | 9.483 | 496.07,300.02,255.03,151.00,125.02 | 3.5359 |
| 52* | kaempferol-3-O-α-L-rhamnopyranoside | C_21_H_20_O_10_ | 431.0982 | 11.343 | 494.09,467.07,284.03,151.00 | 0.3943 |
| 53* | quercetin | C_15_H_10_O_7_ | 301.0341 | 13.103 | 151.00,125.02 | 3.8942 |
| 54* | isoqueritrin | C_21_H_20_O_12_ | 463.0868 | 8.505 | 300.02,255.03,151.00,125.02 | 2.8003 |
| 55* | rutin | C_27_H_30_O_16_ | 609.1458 | 8.183 | 300.02,255.03,151.00,125.02 | -0.4924 |
| 56* | quercetin-3-O-β-D-xyloside | C_20_H_18_O_11_ | 433.0770 | 8.950 | 496.07,300.02,151.00 | 1.4626 |
| 57* | kaempferol-3-O-arabinoside | C_20_H_18_O_10_ | 417.0818 | 10.833 | 480.07,284.03,151.00 | 2.2012 |
| 58* | quercetin-3-O-β-D-glucopyranosyl-(1→2)-β-D-glucopyranoside | C_27_H_30_O_17_ | 625.1395 | 6.674 | 300.02,255.03,151.00,125.02 | 2.4323 |
| 59* | apigenin | C_15_H_10_O_5_ | 269.0457 | 15.384 | 151.00,117.03,107.01 | -0.7433 |
| 60* | luteolin | C_15_H_10_O_6_ | 285.0406 | 13.236 | 151.00,133.03,107.01 | 0.3508 |
| 61* | mangiferin | C_19_H_18_O_11_ | 421.0770 | 5.326 | 305.06 | 1.5042 |
| 62# | 3,4,5-trihydroxy-6-(2-hydroxyethoxy)oxane-2-carboxylic acid | C_8_H_14_O_8_ | 237.0609 | 0.94 | 191.03 | 2.9025 |
| 63# | tricrozarin B | C_13_H_12_O_7_ | 279.0506 | 0.94 | 125.02 | 1.5222 |
| 64# | UNPD230015 | C_13_H_12_O_6_ | 310.0689 | 0.941 | 237.05,165.03,135.02 | 1.9529 |
| 65# | D-galactaro-1,5-lactone(1-) | C_6_H_8_O_7_ | 191.0189 | 0.961 | 128.03,113.02 | 3.7812 |
| 66# | pereflorin B | C_13_H_12_O_7_ | 279.0523 | 0.982 | 125.02 | -4.548 |
| 67# | (2,3,4-trihydroxyphenyl)-(3,4,5-trihydroxyphenyl)methanone | C_13_H_10_O_7_ | 277.0366 | 1.002 | 169.01,125.02 | -4.7611 |
| 68# | D-Glucaro-1,4-lactone | C_6_H_8_O_7_ | 191.0192 | 1.012 | 133.01,113.02 | 2.2190 |
| 69# | succinic acid | C_4_H_6_O_4_ | 117.0188 | 1.032 |  | 4.5092 |
| 70# | D-galactaro-1,5-lactone(1-)-iso | C_6_H_8_O_7_ | 191.0185 | 1.058 | 133.01,111.00 | 5.8643 |
| 71# | D-galactaro-1,5-lactone(1-)-iso | C_6_H_8_O_7_ | 191.0185 | 1.236 | 128.03,111.00 | 5.8643 |
| 72# | beta-Glucogallin | C_13_H_16_O_10_ | 331.0668 | 1.276 | 169.01 | 0.5128 |
| 73# | 1-acyl-3-O-beta-D-galactosyl-sn-glycerol | C_11_H_20_O_9_ | 295.1029 | 1.464 | 147.06 | 0.5558 |
| 74# | beta-Glucogallin-iso | C_13_H_16_O_10_ | 331.0677 | 1.955 | 169.01 | -1.8963 |
| 75# | passiguatemalin | C_12_H_19_NO_8_ | 304.1032 | 2.060 | 147.04 | 1.6063 |
| 76# | UNPD164817 | C_12_H_19_NO_8_ | 304.1028 | 2.338 | 147.04 | -1.0095 |
| 77# | phyllanemblinin D | C_27_H_26_O_20_ | 669.0941 | 2.374 | 337.01 | 0.3981 |
| 78# | phyllanemblinin E | C_27_H_26_O_20_ | 669.0943 | 2.693 | 337.01 | 0.2489 |
| 79# | 3,5-Dihydroxyphenyl 1-O-(6-O-galloyl-beta-D-glucopyranoside) | C_19_H_20_O_12_ | 439.0870 | 3.126 | 313.05 | 2.7258 |
| 80# | 1,6-Digalloyl-beta-D-glucopyranose | C_20_H_20_O_14_ | 483.0776 | 3.358 | 313.05,169.01 | 0.8860 |
| 81# | 1,3,5-trihydroxybenzene 1-O-[4,6-(S)-HHDP]-β-D-glucoside-iso | C_26_H_22_O_16_ | 589.0822 | 3.390 | 300.99,125.02 | -0.1600 |
| 82# | 1,6-Digalloyl-beta-D-glucopyranose-iso | C_20_H_20_O_14_ | 483.0764 | 3.580 | 313.05,169.01 | 3.1583 |
| 83# | 1,6-Digalloyl-beta-D-glucopyranose-iso | C_20_H_20_O_14_ | 483.0775 | 3.779 | 313.05,169.01 | 1.0926 |
| 84# | ellagic acid di-hexoside | C_26_H_26_O_18_ | 625.1032 | 3.946 | 463.04,300.99 | 2.2959 |
| 85# | Com-1 | C_26_H_24_O_17_ | 607.0926 | 4.203 | 275.01,247.02,191.02 | 2.2577 |
| 86# | epigallocatechin gallate | C_22_H_18_O_11_ | 457.0780 | 4.254 | 303.03,169.01 | -0.7970 |
| 87# | amritoside | C_26_H_26_O_18_ | 625.1031 | 4.354 | 463.04,300.99 | -1.5406 |
| 88# | UNPD98846 | C_18_H_21_NO_11_ | 426.1033 | 4.447 | 169.01 | 2.0697 |
| 89# | Com-2 | C_26_H_24_O_17_ | 607.0930 | 4.531 | 273.00,247.02,191.02,169.01 | 1.7643 |
| 90# | UNPD17215 | C_19_H_18_O_12_ | 437.0708 | 4.546 | 275.01,257.00 | 3.7655 |
| 91# | amlaic acid | C_27_H_24_O_19_ | 651.0833 | 4.557 | 437.07,293.02 | 0.7700 |
| 92# | Com-3 | C_26_H_22_O_17_ | 605.0798 | 4.830 | 273.02,169.01 | -2.2723 |
| 93# | pseudolaroside B | C_14_H_18_O_9_ | 329.0872 | 4.487 | 392.08,365.06,275.01,167.03 | 1.8349 |
| 94# | (-)-Epigallocatechin | C_15_H_14_O_7_ | 305.0691 | 5.309 | 341.05 | -8.2454 |
| 95# | methyl neochebulanin | C_28_H_28_O_20_ | 683.1091 | 5.332 | 300.99 | 1.4863 |
| 96# | UNPD162261 | C_18_H_32_O_12_ | 439.1809 | 5.380 | 502.17 | 2.7261 |
| 97# | 1,2,6-Trigalloyl-beta-D-glucopyranose-iso | C_27_H_24_O_18_ | 635.0872 | 5.398 | 465.06,313.05,169.01 | 2.6529 |
| 98# | 1-O-Sinapoylglucose | C_17_H_22_O_10_ | 385.1132 | 5.400 | 169.01 | 1.866 |
| 99# | catechin-iso | C_15_H_14_O_6_ | 289.0711 | 5.518 | 352.06,325.04,179.03,151.03 | 2.2811 |
| 100# | Com-4 | C_25_H_22_O_15_ | 561.0877 | 5.648 | 273.00,247.02 | 1.4119 |
| 101# | SCHEMBL1279584 | C_20_H_16_O_13_ | 463.0515 | 5.670 | 300.99,229.01 | 0.6769 |
| 102# | 1-O-(3-Hydroxybutyryl)pancratistatin | C_18_H_21_NO_10_ | 410.1083 | 5.922 | 271.04,211.02 | 2.1147 |
| 103# | UNPD83927 | C_26_H_24_O_16_ | 591.0977 | 5.940 | 211.02,169.01 | 2.4627 |
| 104# | 1,2,6-Trigalloyl-beta-D-glucopyranose-iso | C_27_H_24_O_18_ | 635.0870 | 6.334 | 465.06,313.05,169.01 | 3.1245 |
| 105# | ent-Epicatechin-(4alpha->8)-ent-epicatechin 3'-gallate | C_37_H_30_O_16_ | 729.1439 | 6.397 | 289.07,169.01 | 2.8876 |
| 106# | strictinin-iso | C_27_H_22_O_18_ | 633.0722 | 6.588 | 463.05,300.99 | 1.7938 |
| 107# | ent-Epicatechin-(4alpha->8)-ent-epicatechin 3-gallate-iso | C_37_H_30_O_16_ | 729.1460 | 6.619 | 792.13,765.12,289.07,169.01 | 0.0115 |
| 108# | kynaComin 13 | C_12_H_10_O_7_ | 265.0340 | 7.104 | 229.01,217.01,177.01 | 4.797 |
| 109# | strictinin-iso | C_27_H_22_O_18_ | 633.0734 | 7.165 | 229.01,217.01,177.01 | -0.2564 |
| 110# | UNPD108351 | C_18_H_23_NO_10_ | 412.1243 | 7.247 | 313.05,169.01 | 1.4995 |
| 111# | UNPD200627 | C_17_H_24_O_10_ | 387.1286 | 7.477 | 450.12,217.00 | 2.5004 |
| 112# | UNPD133818 | C_26_H_22_O_17_ | 605.0785 | 7.589 | 273.00,247.02,191.02,169.01 | -0.2924 |
| 113# | UNPD69168 | C_44_H_34_O_20_ | 881.1577 | 7.824 | 944.15,917.13,407.07,289.06,287.05,169.01 | -0.8309 |
| 114# | Methylol Tannin | C_28_H_26_O_19_ | 665.0994 | 7.862 | 728.09,637.10,300.99,169.01 | -0.3700 |
| 115# | 3,4,5-Trihydroxy-6-({4-Hydroxy-2-Oxo-2H-Furo[2,3-H]Chromen-8-Yl}Oxy)Oxane-2-Carboxylic Acid | C_17_H_14_O_11_ | 393.0443 | 7.924 | 273.00,247.02,191.02 | 5.1638 |
| 116# | tri-O-galloyl-shikimic acid | C_28_H_22_O_17_ | 629.0769 | 8.485 | 477.06,315.01,169.01 | 2.2581 |
| 117# | catechin-3-O-gallate | C_22_H_18_O_10_ | 441.0807 | 8.882 | 289.07,169.01,125.02 | 4.5699 |
| 118# | 6-{[3-({6-[(Acetyloxy)Methyl]-3,4,5-Trihydroxyoxan-2-Yl}Oxy)-5-Hydroxy-4-Oxo-2-(3,4,5-Trihydroxyphenyl)-4H-Chromen-7-Yl]Oxy}-3,4,5-Trihydroxyoxane-2-Carboxylic Acid | C_29_H_30_O_20_ | 697.1248 | 8.882 | 665.09,637.10,300.02,169.01 | 1.385 |
| 119# | Com-5 | C_25_H_36_O_15_ | 575.1970 | 9.202 | 638.19,611.17 | 1.9854 |
| 120# | polydine | C_20_H_22_O_10_ | 421.1129 | 9.357 | 484.10,169.01 | 2.6545 |
| 121# | (-)-Epiafzelechin 3-gallate | C_22_H_18_O_9_ | 425.0885 | 9.967 | 488.08,169.01 | -1.8641 |
| 122# | 3,5-Digalloylepicatechin | C_29_H_22_O_14_ | 593.0924 | 10.422 | 289.07,169.01 | 2.1527 |
| 123# | SCHEMBL17220937 | C_27_H_24_O_16_ | 603.0982 | 11.392 | 431.09,300.99 | 1.4206 |
| 124# | 3,5-Digalloylepicatechin | C_29_H_22_O_14_ | 593.0911 | 11.412 | 407.07,285.03 | 4.1725 |
| 125# | (+)-Pinoresinol 4-O-(6''-O-galloyl)-beta-D-glucopyranoside | C_33_H_36_O_15_ | 671.1972 | 11.467 | 314.04 | 1.4043 |
| 126# | Com-6 | C_27_H_24_O_16_ | 603.0990 | 11.776 | 431.09,300.99 | 0.2619 |
| 127# | Com-7 | C_28_H_24_O_18_ | 647.0879 | 12.578 | 435.05,273.00,247.02,167.03 | 1.5237 |
| 128# | Com-8 | C_28_H_22_O_17_ | 629.0778 | 12.970 | 692.07,435.05,298.98,273.00,247.02,167.03 | 0.9884 |
| 129# | Com-9 | C_29_H_26_O_18_ | 661.1040 | 14.002 | 724.09,697.07,273.00,247.02 | -1.76 |
| 130# | Com-10 | C_28_H_28_O_17_ | 635.1250 | 14.248 | 603.09,435.05,273.00,247.02 | 0.4292 |
| 131# | quercetin 3-O-glucuronide | C_21_H_18_O_13_ | 477.0666 | 7.822 | 302.00,257.00 | 1.5985 |
| 132# | kaempferol 3-rhamnoside-7-glucoside | C_27_H_30_O_15_ | 593.1520 | 8.610 | 351.03,284.03,149.01 | -1.3569 |
| 133# | trilobatin | C_21_H_24_O_10_ | 435.1290 | 10.823 | 498.12,273.07 | 1.5374 |
| 134# | trilobatin-iso | C_21_H_24_O_10_ | 435.1291 | 12.473 | 273.07,167.03 | 1.3081 |
| 135# | aromadendrin | C_15_H_12_O_6_ | 287.0553 | 12.691 | 151.00,125.02 | 2.4706 |
| 136# | sakuranin | C_22_H_24_O_10_ | 447.1281 | 12.928 | 285.07,165.01 | 3.5045 |
| 137# | pinocembrin-5-O-[3′′-O-galloyl]-β-D-glucose | C_21_H_22_O_9_ | 569.1284 | 15.909 | 632.12,313.05,255.06,169.01 | 2.9193 |
| 138# | chalcone-7-O-β-D-glucopyranoside | C_21_H_22_O_9_ | 417.1189 | 17.472 | 255.06 | 0.2530 |
| 139# | pinocembrin-7-O-[2′′-O-galloyl]-β-D-glucose | C_28_H_26_O_13_ | 569.1298 | 17.942 | 313.05,255.06,169.01 | 0.2884 |
| 140# | pinostrobin 5-glucoside | C_22_H_24_O_9_ | 431.1331 | 19.472 | 494.13,477.14,269.08,165.01 | 3.8318 |
| 141# | chalcone-7-O-[3′′-O-galloyl]-β-D-glucose | C_28_H_26_O_13_ | 569.1302 | 20.026 | 273.00,255.06,169.01 | -0.2378 |
| 142# | chalcone-7-O-[2′′-O-galloyl]-β-D-glucose | C_28_H_26_O_13_ | 569.1294 | 20.269 | 273.00,255.06,169.01 | 0.9899 |
| 143# | 4',5,7-Trimethoxyflavone | C_18_H1_6_O_5_ | 311.0914 | 21.896 | 374.08 | 3.5155 |
| 144# | 1-(2,6-Dihydroxy-4-methoxyphenyl)-3-phenyl-1-propanone | C_16_H_16_O_4_ | 271.0962 | 28.076 | 152.00,139.04,125.02 | 1.2825 |
| 145# | Com-11 | C_36_H_28_O_16_ | 715.1267 | 27.334 | 273.00 | 5.2482 |
| 146# | Com-12 | C_35_H_30_O_18_ | 737.1360 | 15.622 | 300.99,255.06 | -0.0844 |
| 147# | chalcone-7-O-[4′′,6′′-(S)-hexahydroxydiphenoyl]-β-D-glucoside | C_35_H_28_O_17_ | 719.1252 | 21.415 | 300.99,255.06 | 0.2402 |
| 148# | chalcone-5-O-[4′′,6′′-(S)-hexahydroxydiphenoyl]-β-D-glucoside | C_35_H_30_O_17_ | 721.1411 | 22.092 | 300.99,255.06 | -0.1066 |
| 149# | Com-13 | C_36_H_32_O_17_ | 735.1537 | 23.478 | 300.99,257.08 | 4.0386 |
| 150# | pinocembrin-5-O-[4′′,6′′-(S)-hexahydroxydiphenoyl]-β-D-glucoside | C_36_H_30_O_17_ | 733.1391 | 24.047 | 300.99,269.08 | 2.3469 |
| 151# | alpinetin-7-O-[4′′,6′′-(S)-hexahydroxydiphenoyl]-β-D-glucoside | C_36_H_30_O_17_ | 733.1393 | 24.333 | 300.99,269.08 | 2.3469 |
| 152# | Com-14 | C_36_H_32_O_17_ | 735.1554 | 25.209 | 300.99,271.09 | 1.7294 |
| 153# | Com-15 | C_42_H_32_O_21_ | 871.1339 | 26.093 | 300.99,255.06,169.01 | 2.6734 |
| 154# | alpinetin-7-O-[3′′-O-galloyl-4′′,6′′-(S)-hexahydroxydiphenoyl]-β-D-glucoside | C_43_H_34_O_21_ | 885.1508 | 27.009 | 583.14,300.99,269.08,169.01 | 0.5400 |
| 155# | Com-16 | C_36_H_34_O_17_ | 737.1710 | 27.326 | 435.05,300.99 | 1.7925 |
| 156# | Com-17 | C_43_H_36_O_21_ | 887.1661 | 27.438 | 585.15,300.99,271.09,169.01 | 1.7245 |
| 157# | alpinetin-7-O-[2′′-O-galloyl-4′′,6′′-(S)-hexahydroxydiphenoyl]-β-D-glucoside | C_43_H_34_O_21_ | 885.1513 | 27.796 | 300.99,269.08,169.0 | 0.77 |
| 158# | 5'-β-D-glucopyranosyloxyjasmonic acid-iso | C_18_H_28_O_9_ | 387.1651 | 5.374 | 450.16 | 2.4628 |
| 159# | 5'-β-D-glucopyranosyloxyjasmonic acid | C_18_H_28_O_9_ | 387.1649 | 5.817 | 450.16,225.10 | 2.9781 |
| 160# | gingerol | C_17_H_26_O_4_ | 293.1743 | 24.386 | 329.15,221.15 | 5.2107 |
| 161# | gingerglycolipid A | C_33_H_56_O_14_ | 675.3572 | 29.187 | 738.35,711.33,397.13 | 3.7406 |
| 162# | (9S,10S)-9,10-dihydroxyoctadecanoate | C_18_H_36_O_4_ | 315.2536 | 31.764 | 277.21 | 1.5279 |
| 163# | UNPD217236 | C_21_H_40_O_6_ | 387.2751 | 31.932 | 325.18,277.21 | 0.2897 |
| 164# | betulinic acid | C_30_H_48_O_3_ | 455.3525 | 32.296 | 309.32 | 1.2466 |
| 165# | alpha-Linolenic acid | C_18_H_30_O_2_ | 277.2164 | 32.571 | 251.16,211.13 | 3.2481 |
| 166# | 2-hydroxyl-28-O-[α-L-rhamnopyranosyl-(1→2)-β-D-glucopyranoside]-ursolic acid | C_42_H_68_O_11_ | 747.4711 | 32.784 | 277.21 | -2.9573 |
| 167# | linoleic acid | C_18_H_32_O_2_ | 279.2316 | 33.333 | 255.23 | 4.474 |
| 168# | soyacerebroside I | C_40_H_75_NO_9_ | 712.5372 | 33.441 | 775.53,748.51,532.47,473.28 | -0.5515 |
| 169# | vaccenic acid | C_18_H_34_O_2_ | 281.2477 | 34.136 | 277.21,255.23 | 3.2024 |

Note: "*" was the comparative chemical composition of licorice compound library, "#" was the presumed chemical composition
